# Factors Associated With Nonadherence to Lung Cancer Screening Across Multiple Screening Time Points

**DOI:** 10.1001/jamanetworkopen.2023.15250

**Published:** 2023-05-25

**Authors:** Yannan Lin, Li-Jung Liang, Ruiwen Ding, Ashley Elizabeth Prosper, Denise R. Aberle, William Hsu

**Affiliations:** 1 Department of Bioengineering, University of California, Los Angeles; 2 Medical & Imaging Informatics, Department of Radiological Sciences, David Geffen School of Medicine at UCLA, Los Angeles, California; 3 Department of Medicine, University of California, Los Angeles

## Abstract

**Importance:**

Screening with low-dose computed tomography (CT) has been shown to reduce mortality from lung cancer in randomized clinical trials in which the rate of adherence to follow-up recommendations was over 90%; however, adherence to Lung Computed Tomography Screening Reporting & Data System (Lung-RADS) recommendations has been low in practice. Identifying patients who are at risk of being nonadherent to screening recommendations may enable personalized outreach to improve overall screening adherence.

**Objective:**

To identify factors associated with patient nonadherence to Lung-RADS recommendations across multiple screening time points.

**Design, Setting, and Participants:**

This cohort study was conducted at a single US academic medical center across 10 geographically distributed sites where lung cancer screening is offered. The study enrolled individuals who underwent low-dose CT screening for lung cancer between July 31, 2013, and November 30, 2021.

**Exposures:**

Low-dose CT screening for lung cancer.

**Main Outcomes and Measures:**

The main outcome was nonadherence to follow-up recommendations for lung cancer screening, defined as failing to complete a recommended or more invasive follow-up examination (ie, diagnostic dose CT, positron emission tomography–CT, or tissue sampling vs low-dose CT) within 15 months (Lung-RADS score, 1 or 2), 9 months (Lung-RADS score, 3), 5 months (Lung-RADS score, 4A), or 3 months (Lung-RADS score, 4B/X). Multivariable logistic regression was used to identify factors associated with patient nonadherence to baseline Lung-RADS recommendations. A generalized estimating equations model was used to assess whether the pattern of longitudinal Lung-RADS scores was associated with patient nonadherence over time.

**Results:**

Among 1979 included patients, 1111 (56.1%) were aged 65 years or older at baseline screening (mean [SD] age, 65.3 [6.6] years), and 1176 (59.4%) were male. The odds of being nonadherent were lower among patients with a baseline Lung-RADS score of 1 or 2 vs 3 (adjusted odds ratio [AOR], 0.35; 95% CI, 0.25-0.50), 4A (AOR, 0.21; 95% CI, 0.13-0.33), or 4B/X, (AOR, 0.10; 95% CI, 0.05-0.19); with a postgraduate vs college degree (AOR, 0.70; 95% CI, 0.53-0.92); with a family history of lung cancer vs no family history (AOR, 0.74; 95% CI, 0.59-0.93); with a high age-adjusted Charlson Comorbidity Index score (≥4) vs a low score (0 or 1) (AOR, 0.67; 95% CI, 0.46-0.98); in the high vs low income category (AOR, 0.79; 95% CI, 0.65-0.98); and referred by physicians from pulmonary or thoracic-related departments vs another department (AOR, 0.56; 95% CI, 0.44-0.73). Among 830 eligible patients who had completed at least 2 screening examinations, the adjusted odds of being nonadherent to Lung-RADS recommendations at the following screening were increased in patients with consecutive Lung-RADS scores of 1 to 2 (AOR, 1.38; 95% CI, 1.12-1.69).

**Conclusions and Relevance:**

In this retrospective cohort study, patients with consecutive negative lung cancer screening results were more likely to be nonadherent with follow-up recommendations. These individuals are potential candidates for tailored outreach to improve adherence to recommended annual lung cancer screening.

## Key Points

**Question:**

What factors and longitudinal patterns are associated with patient nonadherence to lung cancer screening across multiple screening time points?

**Findings:**

In this cohort study of 1979 patients, 6 factors, including baseline Lung Computed Tomography Screening Reporting & Data System (Lung-RADS) score, were found to be associated with patient nonadherence to recommended follow-up examination. Nonadherence increased over time for patients who received consecutive Lung-RADS scores of 1 or 2.

**Meaning:**

The findings suggest that patients with consecutive negative screening results (Lung-RADS score, 1 or 2) are more likely to become nonadherent to screening over time and may benefit from outreach and education.

## Teaser

This cohort study investigates factors associated with patient nonadherence to lung cancer screening recommendations across multiple time points.

## Introduction

Screening with low-dose computed tomography (LDCT) effectively reduced mortality from lung cancer by at least 20% in large randomized clinical trials in which adherence rates were over 90%.^1,2^ The Lung CT Screening Reporting & Data System (Lung-RADS), released in 2014, has become a nationally accepted standard for lung cancer screening (LCS) CT reporting and management recommendations.^3^ The follow-up recommendations are to continue annual LDCT screening in patients with Lung-RADS scores of 1 or 2; in patients with Lung-RADS scores of 3 or 4, early or more aggressive follow-up is advised, which may entail LDCT in 6 months, LDCT in 3 months, chest CT, positron emission tomography–CT (PET-CT), or tissue sampling.^4^ Notably, patient adherence to LCS in clinical practice is substantially lower than the over 90% adherence rates in clinical trials. A systematic review and meta-analysis^5^ by some of us found that patient adherence to baseline Lung-RADS recommendations was 57% to 65% in clinical LCS programs, with a significantly lower annual adherence rate among patients with Lung-RADS scores of 1 or 2 (45%-49%) compared with early follow-up adherence among those with Lung-RADS scores of 3 or 4 (74%-78%). Similarly, a recent study reported that rates of adherence to recommendations from baseline and the first annual screening were 48% and 44%, respectively, among patients with Lung-RADS scores of 1 or 2 in a large national cohort (N = 30 166).^6^ Failing to maintain annual adherence to LCS recommendations may diminish the ability of clinical screening programs to achieve the same mortality benefits found in large clinical trials. Interval lung cancers, diagnosed between screening episodes following a preceding negative screening result (Lung-RADS score, 1 or 2), are more likely to be aggressive, emphasizing the importance of regular screening intervals.^7^

Lung cancer screening is nascent as a preventive measure in the US; as such, barriers to LCS have been incompletely investigated. Patient-level barriers include unawareness of screening benefits and risks; perceptual barriers, such as fear of cancer diagnosis and perceived stigma; screening-related cost concerns; and challenges in accessing imaging sites.^8^ Identifying factors that affect patient adherence to Lung-RADS recommendations can help clinicians better understand who would benefit from outreach strategies to improve adherence.^9^ These factors may be used to identify patients who are at risk for nonadherence. Given that patient characteristics in clinical LCS programs vary across institutions, clinical risk stratification models that aid in the identification of potentially nonadherent patients may result in more aggressive, tailored approaches and thus improve the mortality benefit of screening. To our knowledge, no studies have investigated factors associated with patient nonadherence to Lung-RADS recommendations over multiple screening intervals. Specifically, Lung-RADS scores may vary over time. Previous work has shown that the Lung-RADS score was a significant factor associated with nonadherence to baseline Lung-RADS recommendations^5^; however, evidence on whether longitudinal patterns of Lung-RADS scores affect the risk of nonadherence to screening in the future is lacking.

This study aimed to identify factors associated with risk for patient nonadherence to Lung-RADS recommendations at baseline and across multiple time points. In experiment 1, we investigated whether patient demographics, socioeconomic status, and health status were associated with nonadherence to baseline Lung-RADS recommendations. Experiment 2 adjusted for significant factors from experiment 1 to evaluate the hypothesis that adherence would increase or decrease as Lung-RADS scores were upgraded or downgraded, respectively, and adherence would be stable when Lung-RADS scores remained unchanged.

## Methods

### Patient Enrollment

Institutional review board approval was obtained from the University of California, Los Angeles (UCLA), to conduct this retrospective cohort study, and informed consent was waived because the risk to participants was minimal. We included patients who underwent at least 1 LDCT screening examination at our institution from July 31, 2013, to November 30, 2021 (last follow-up, December 8, 2021), within 10 geographically distributed sites where LCS is offered. Lung-RADS scores were retrospectively assigned to LDCT screenings performed prior to the release date of Lung-RADS version 1.0^10^ by a board-certified thoracic radiologist (D.R.A.). Patient exclusion criteria are summarized in the Figure. Annual screenings or early follow-up LDCT screenings were excluded if patients were older than 80 years at the time of screening, the patient had a Lung-RADS score of 0, or the screening was incorrectly ordered for nonscreening purposes. Additional details are reported in eAppendix 1 in Supplement 1, such as identification of screening-eligible patients and intervention for adherence. This study followed the Strengthening the Reporting of Observational Studies in Epidemiology (STROBE) reporting guideline for cohort studies.

**Figure.**
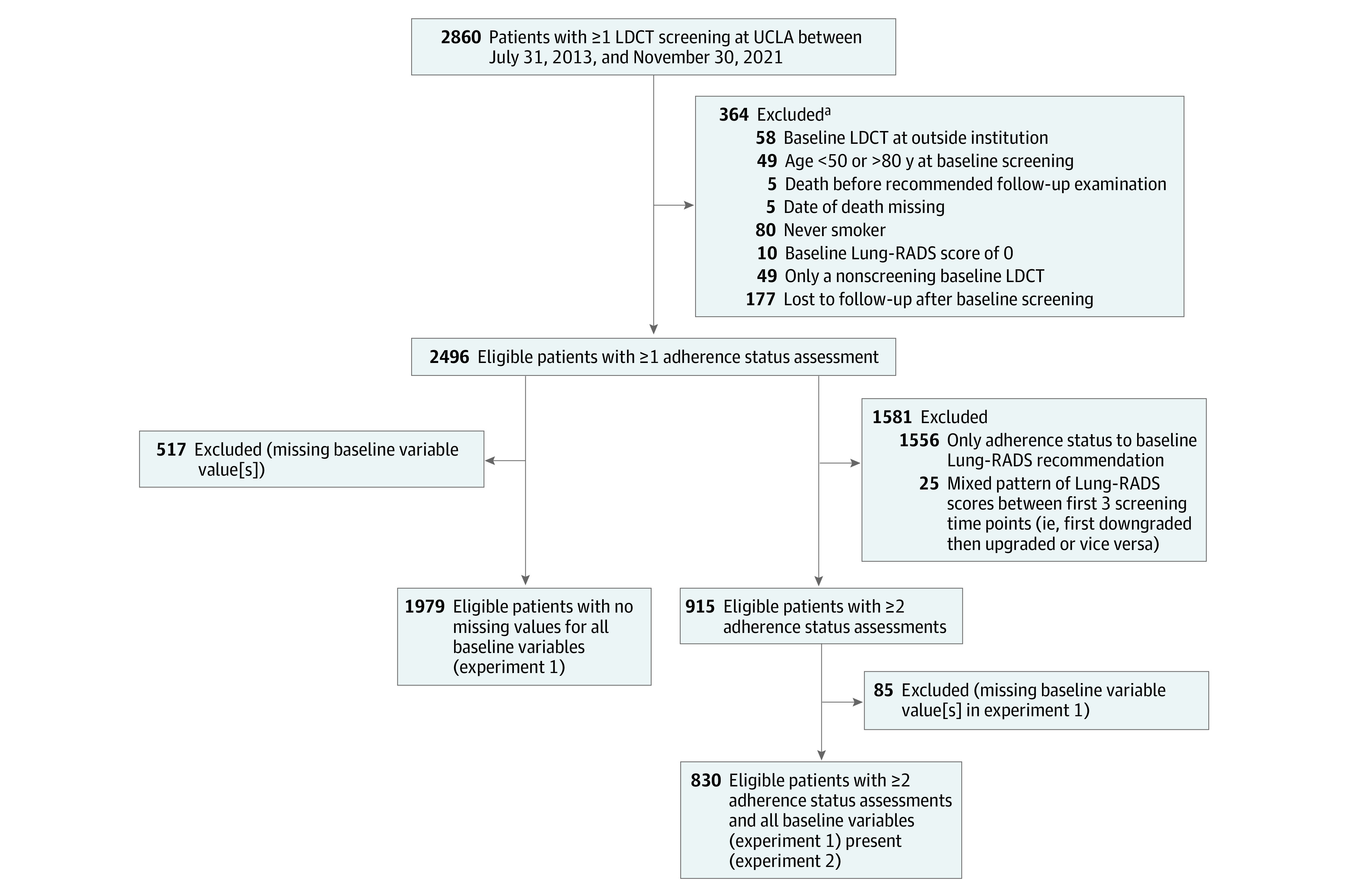
Flow Diagram of Patient Enrollment The last follow-up was December 8, 2021. CT indicates computed tomography; LDCT, low-dose CT; Lung-RADS, Lung CT Screening Reporting & Data System; UCLA, University of California, Los Angeles. ^a^A patient could have more than 1 exclusion criterion.

### Data Collection

Patient characteristics at the time of their baseline screening were obtained by medical abstraction from our institution’s electronic health record, including an existing registry of patients who undergo LCS. Baseline factors of interest included Lung-RADS score, age, sex, race and ethnicity, educational level, family history of lung cancer, smoking status, primary insurance status, age-adjusted Charlson Comorbidity Index (CCI) score,^11^ distance to screening center, median family income, area deprivation index (ADI) state rank,^12^ and type of referring physician. Race and ethnicity data (included to assess the comparative racial and ethnic proportions of UCLA patients undergoing screening and because differences in risk of lung cancer have been identified across different racial and ethnic groups) were obtained from a self-reported questionnaire administered prior to the LDCT screening examination that was stored as a discrete series of the screening examination in our picture archiving and communication system. When such data were missing from the questionnaire, data in the electronic medical record were extracted. Race and ethnicity categories included Asian, Black, Hispanic or Latino, White, and other (American Indian or Alaska Native, Native Hawaiian or Pacific Islander, more than 1 race, or racial and ethnic group not otherwise stated). Age-adjusted CCI score was grouped into 3 categories: low (0 or 1), intermediate (2 or 3), and high (≥4).^11^ Median family income was mapped with the 2010 census data using the home zip code. Distance to screening center was estimated between the home zip code and the zip code of the screening center. We dichotomized the variables ADI state rank, median family income, and distance to screening center as low or short (less than or equal to the median) and high or long (greater than the median). Elective imaging examinations, such as LDCT screening examinations, were suspended at the beginning of the COVID-19 pandemic (ie, from March 19 to May 19, 2020) at our institution to conserve health care resources and minimize the risk of viral transmission. To account for the potential association of this pause in elective imaging examinations with patient adherence, we included 1 variable indicating whether or not the date of the expected follow-up examination fell within the 2-month pause period.

### Patient Outcomes

The main outcome of the study was patient nonadherence, defined as failing to adhere to follow-up recommendations based on Lung-RADS category, factoring in some time allowance from the recommended period. Adherence was defined for a Lung-RADS score of 1 or 2 as completing the next annual screening within 12 months plus 3 months, for a Lung-RADS score of 3 as completing a recommended repeated LDCT within 6 months plus 3 months, for a Lung-RADS score of 4A as completing an LDCT within 3 months plus 2 months, and for a Lung-RADS score of 4B/X as completing a more aggressive diagnostic workup (ie, diagnostic chest CT, PET-CT, or tissue sampling) within 3 months of abnormal screening findings (eFigure 1 in Supplement 1). Patients were considered adherent if they completed a more invasive follow-up examination (ie, diagnostic chest CT, PET-CT, or tissue sampling vs LDCT) within the defined time intervals.

### Statistical Analysis

In experiment 1, we used a multivariable logistic regression model to identify factors significantly associated with nonadherence to baseline Lung-RADS recommendations. Patients who had missing values in some characteristics were excluded from the analysis. A sensitivity analysis was implemented using multiple imputation data (ie, the mice^13^ package in R, version 3.6.1 [R Project for Statistical Computing]) and found similar results.

In experiment 2, we examined whether the baseline Lung-RADS score and a pattern of subsequent Lung-RADS scores were associated with nonadherence to Lung-RADS recommendations over time. Patients who underwent at least 2 screening examinations were included in this analysis. The Lung-RADS score was binary (1 or 2 vs 3 or 4). A Lung-RADS score of 1 or 2 was defined as a negative screening result, and a Lung-RADS score of 3 or 4 was defined as a positive screening result. Patients were categorized into subgroups based on their longitudinal patterns of Lung-RADS scores (eTable 2 in Supplement 1): unchanged, upgraded (negative to positive), or downgraded (positive to negative). Patients whose Lung-RADS scores were first upgraded and then downgraded or vice versa were excluded. These patients may have had more complex changes in health status (eg, first upgraded, then downgraded: health status worsened, then improved) than those with monotonic or no changes in Lung-RADS scores (eg, downgraded: health status improved). A generalized estimating equations (GEE) model with a logit link and an unstructured working correlation that accounted for repeated measurements within the same patient was used. The fixed effects included in this model were baseline Lung-RADS score (1 or 2 vs 3 or 4), longitudinal patterns of Lung-RADS scores (changed vs unchanged), screening time point (first, second, or third), three 2-way interaction terms, one 3-way interaction term among the 3 variables, and significant baseline factors associated with nonadherence from experiment 1 (ie, *z* test 2-sided *P* <  .05). Patients who had missing values for some factors were excluded from this analysis. Python, version 3.7.3 (Python Software Foundation) and R, version 3.6.1^14^ were used for data analyses. Two-sided *P* < .05 was considered significant.

## Results

Among the 2496 eligible patients, 1979 had no missing values in all baseline characteristics (Figure). A comparison of the observed baseline characteristics between included and excluded patients is shown in eTable 1 in Supplement 1. No significant differences were found for any variables except family history of lung cancer. The majority of patients (1660 [83.9%]) had a negative baseline screening result and were 65 years of age or older (1111 [56.1%]). Mean (SD) age was 65.3 (6.6) years; 803 patients (40.6%) were female; 1176 (59.4%), male; 169 (8.5%), Asian; 130 (6.6%), Black; 111 (5.6%), Hispanic or Latino; 1526 (77.1%), White; and 43 (2.2%), other race and ethnicity. A total of 1210 patients were former smokers (61.1%). Patient characteristics at the baseline screening are summarized in Table 1. The mean follow-up time was 1.78 years (range, 0.25-3.75 years) (eTable 4 in Supplement 1 shows details). Eighty-one of the 2496 eligible patients (3.2%) were diagnosed with primary lung cancers during follow-up.

**Table 1.** Baseline Patient Characteristics in Experiment 1

Variable	Patients, No. (%)		
	Overall (N = 1979)	Adherent to LCS (n = 693)	Nonadherent to LCS (n = 1286)
Lung-RADS score			
1 or 2	1660 (83.9)	490 (70.7)	1170 (91.0)
3	154 (7.8)	83 (12.0)	71 (5.5)
4A	99 (5.0)	67 (9.7)	32 (2.5)
4B/X	66 (3.3)	53 (7.6)	13 (1.0)
Age, y			
<65	868 (43.9)	268 (38.7)	600 (46.7)
≥65	1111 (56.1)	425 (61.3)	686 (53.3)
Sex			
Female	803 (40.6)	276 (39.8)	527 (41.0)
Male	1176 (59.4)	417 (60.2)	759 (59.0)
Race and ethnicity			
Asian	169 (8.5)	59 (8.5)	110 (8.6)
Black	130 (6.6)	49 (7.1)	81 (6.3)
Hispanic or Latino	111 (5.6)	35 (5.1)	76 (5.9)
White	1526 (77.1)	540 (77.9)	986 (76.7)
Other^a^	43 (2.2)	10 (1.4)	33 (2.6)
Educational level			
Less than college	958 (48.4)	337 (48.6)	621 (48.3)
College graduate	590 (29.8)	186 (26.8)	404 (31.4)
Postgraduate	431 (21.8)	170 (24.5)	261 (20.3)
Family history of lung cancer			
Yes	466 (23.5)	187 (27.0)	279 (21.7)
No	1513 (76.5)	506 (73.0)	1007 (78.3)
Smoking status			
Current	769 (38.9)	246 (35.5)	523 (40.7)
Former	1210 (61.1)	447 (64.5)	763 (59.3)
Primary insurance			
Medicare and/or Medicaid	830 (41.9)	328 (47.3)	502 (39.0)
Private or commercial	1121 (56.6)	358 (51.7)	763 (59.3)
Other^b^	28 (1.4)	7 (1.0)	21 (1.6)
Age-adjusted CCI score			
0 or 1	287 (14.5)	72 (10.4)	215 (16.7)
2 or 3	1152 (58.2)	403 (58.2)	749 (58.2)
≥4	540 (27.3)	218 (31.5)	322 (25.0)
Distance to screening center^c^			
≤Median	994 (50.2)	346 (49.9)	648 (50.4)
>Median	985 (49.8)	347 (50.1)	638 (49.6)
Household income^d^			
≤Median	1029 (52.0)	340 (49.1)	689 (53.6)
>Median	950 (48.0)	353 (50.9)	597 (46.4)
ADI state rank^e^			
≤Median	1072 (54.2)	387 (55.8)	685 (53.3)
>Median	907 (45.8)	306 (44.2)	601 (46.7)
Type of referring physician			
Pulmonology, thoracic oncology, radiology, or surgery	369 (18.6)	176 (25.4)	193 (15.0)
Other^f^	1610 (81.4)	517 (74.6)	1093 (85.0)
Expected follow-up examination			
Before COVID-19	1468 (74.2)	513 (74.0)	955 (74.3)
During COVID-19 pause	53 (2.7)	11 (1.6)	42 (3.3)
After COVID-19 pause	458 (23.1)	169 (24.4)	289 (22.5)

Abbreviations: ADI, Area Deprivation Index; CCI, Charlson Comorbidity Index; LCS, lung cancer screening; Lung-RADS, Lung CT Screening Reporting & Data System.

^a^ Subcategories were American Indian or Alaska Native, Native Hawaiian or Pacific Islander, more than 1 race, or racial and ethnic group not otherwise stated.

^b^ Subcategories were Veterans Health Administration (n = 1), self-pay (n = 27), and other insurance not specified (n = 1).

^c^ Median distance to a screening center was 6.84 miles.

^d^ Median household income was $73 478.

^e^ Median ADI state rank was 3.

^f^ Subcategories were family medicine, general internal medicine, and obstetrics and gynecology.

### Lung-RADS Score at Baseline and Nonadherence to Baseline Lung-RADS Recommendations

Among the 1979 patients, the rates of nonadherence to baseline Lung-RADS recommendations were 70.5% (1170 of 1660), 46.1% (71 of 154), 32.3% (32 of 99), and 19.7% (13 of 66) for patients with Lung-RADS scores of 1 or 2, 3, 4A, and 4B/X, respectively. The odds of being nonadherent were lower among patients with a positive baseline Lung-RADS score compared with those with a negative baseline score: for a score of 3, the adjusted odds ratio (AOR) was 0.35 (95% CI, 0.25-0.50); 4A, 0.21 (95% CI, 0.13-0.33); and 4B/X, 0.10 (95% CI, 0.05-0.19) (Table 2). Lower odds of nonadherence were also observed among patients with a postgraduate degree vs a college degree (AOR, 0.70; 95% CI, 0.53-0.92), with family history of lung cancer vs no family history (AOR, 0.74; 95% CI, 0.59-0.93), with a high age-adjusted CCI score (≥4) vs a low score (0 or 1) (AOR, 0.67; 95% CI, 0.46-0.98), in the high vs low income category (AOR, 0.79; 95% CI, 0.65-0.98), and referred by physicians from pulmonary or thoracic-related departments (ie, thoracic oncology, radiology, or surgery) vs another department (AOR, 0.56; 95% CI, 0.44-0.73). These factors were used as inputs into multiple machine learning classifiers to predict patient nonadherence, with the top-performing model achieving a sensitivity of 0.94, specificity of 0.71, and accuracy of 0.72 on the hold-out test data (eAppendix 2, eFigure 2, and eTable 3 in Supplement 1).

**Table 2.** Multivariable Logistic Regression Analysis of Patient Nonadherence to Baseline Lung-RADS Recommendations Among 1979 Patients in Experiment 1

Variable	AOR (95% CI)	*P* value
Intercept	9.17 (4.14-21.65)	<.001
Lung-RADS score		
1 or 2	1 [Reference]	NA
3	0.35 (0.25-0.50)	<.001
4A	0.21 (0.13-0.33)	<.001
4B/X	0.10 (0.05-0.19)	<.001
Age, y		
<65	1 [Reference]	NA
≥65	1.00 (0.78-1.28)	.98
Sex		
Female	1 [Reference]	NA
Male	0.95 (0.77-1.16)	.60
Race and ethnicity		
Asian	0.98 (0.69-1.41)	.90
Black	0.84 (0.56-1.25)	.37
Hispanic or Latino	1.10 (0.71-1.73)	.67
White	1 [Reference]	NA
Other^a^	1.55 (0.77-3.39)	.24
Educational level		
Less than college	0.88 (0.69-1.11)	.28
College graduate	1 [Reference]	NA
Postgraduate	0.70 (0.53-0.92)	.01
Smoking status		
Current smoker	1 [Reference]	NA
Former smoker	0.84 (0.68-1.03)	.10
Family history of lung cancer		
Yes	0.74 (0.59-0.93)	.01
No	1 [Reference]	NA
Primary insurance		
Medicare and/or Medicaid	1 [Reference]	NA
Private or commercial	1.10 (0.88-1.38)	.41
Other^b^	1.41 (0.60-3.70)	.46
Age-adjusted CCI score		
0 or 1	1 [Reference]	NA
2 or 3	0.73 (0.52-1.02)	.07
≥4	0.67 (0.46-0.98)	.04
Distance to screening center		
≤50th Percentile	1 [Reference]	NA
>50th Percentile	1.01 (0.81-1.25)	.95
ADI state rank		
≤50th Percentile	1 [Reference]	NA
>50th Percentile	1.12 (0.90-1.40)	.30
Median annual income		
≤50th Percentile	1 [Reference]	NA
>50th Percentile	0.79 (0.65-0.98)	.03
Type of referring physician		
Pulmonology, thoracic oncology, radiology, or surgery	0.56 (0.44-0.73)	<.001
Other^c^	1 [Reference]	NA
Expected follow-up examination		
Before COVID-19	0.56 (0.27-1.08)	.10
During COVID-19 pause	1 [Reference]	NA
After COVID-19 pause	0.52 (0.24-1.02)	.07

Abbreviations: ADI, Area Deprivation Index; AOR, adjusted odds ratio; CCI, Charlson Comorbidity Index; Lung-RADS, Lung CT Screening Reporting & Data System; NA, not applicable.

^a^ Subcategories were American Indian or Alaska Native, Native Hawaiian or Pacific Islander, more than 1 race, or racial and ethnic group not otherwise stated.

^b^ Subcategories were Veterans Health Administration, self-pay, and other insurance not specified.

^c^ Subcategories were family medicine, general internal medicine, and obstetrics and gynecology.

### Consecutive Negative Screening Results and Nonadherence at Follow-up Screening

A total of 915 patients had 2 or 3 adherence status assessments and monotonic changes in Lung-RADS scores over time; 830 of them (90.7%) had no missing values in all baseline factors significantly associated with adherence in experiment 1 (Figure). No significant differences in the observed variables were found between the included and excluded patients. Most patients (657 [79.2%]) were in the unchanged category (631 [96.0%] negative, 26 [4.0%] positive); 94 of 830 (11.3%) and 79 of 830 (9.5%) were in the downgraded and upgraded categories, respectively. Patient baseline characteristics stratified by patterns of subsequent Lung-RADS scores are shown in Table 3. In the group with unchanged negative screening results compared with the other 3 groups combined, fewer patients were aged 65 years or older (338 of 631 [53.6%] vs 132 of 199 [66.3%]; *P* = .002) and were referred by pulmonary medicine or thoracic-related subspecialists (102 of 631 [16.2%] vs 48 of 199 [24.1%]; *P* = .01).

**Table 3.** Characteristics at Baseline Among 830 Patients, Stratified by Changes in Lung-RADS Scores Across 3 Screening Time Points in Experiment 2

Characteristic	Patients, No. (%)			
	Negative unchanged results (n = 631)	Positive unchanged results (n = 26)	Lung-RADS downgraded (n = 94)	Lung-RADS upgraded (n = 79)
Lung-RADS score^a^				
1 or 2	631 (100)	0	0	79 (100)
3 or 4	0	26 (100)	94 (100)	0
Age, y^b^				
<65	293 (46.4)	5 (19.2)	37 (39.4)	25 (31.6)
≥65	338 (53.6)	21 (80.8)	57 (60.6)	54 (68.4)
Sex				
Female	250 (39.6)	10 (38.5)	33 (35.1)	36 (45.6)
Male	381 (60.4)	16 (61.5)	61 (64.9)	43 (54.4)
Race and ethnicity				
Asian	56 (8.9)	2 (7.7)	10 (10.6)	5 (6.3)
Black	46 (7.3)	2 (7.7)	5 (5.3)	5 (6.3)
Hispanic or Latino	27 (4.3)	1 (3.8)	6 (6.4)	5 (6.3)
White	472 (74.8)	20 (76.9)	69 (73.4)	61 (77.2)
Other^c^	16 (2.5)	0	2 (2.1)	2 (2.5)
Missing	14 (2.2)	1 (3.8)	2 (2.1)	1 (1.3)
Educational level^a^				
Less than college	281 (44.5)	17 (65.4)	42 (44.7)	43 (54.4)
College graduate	196 (31.1)	5 (19.2)	34 (36.2)	19 (24.1)
Postgraduate	154 (24.4)	4 (15.4)	18 (19.1)	17 (21.5)
Smoking status				
Current	253 (40.1)	7 (26.9)	42 (44.7)	37 (46.8)
Former	364 (57.7)	19 (73.1)	52 (55.3)	41 (51.9)
Missing	14 (2.2)	0	0	1 (1.3)
Family history of lung cancer^a^				
Yes	140 (22.2)	4 (15.4)	20 (21.3)	21 (26.6)
No	491 (77.8)	22 (84.6)	74 (78.7)	58 (73.4)
Age-adjusted CCI score^a^				
0 or 1	84 (13.3)	2 (7.7)	9 (9.6)	6 (7.6)
2 or 3	407 (64.5)	16 (61.5)	58 (61.7)	48 (60.8)
≥4	140 (22.2)	8 (30.8)	27 (28.7)	25 (31.6)
Primary insurance				
Medicare and/or Medicaid	272 (43.1)	18 (69.2)	47 (50.0)	30 (38.0)
Private or commercial	348 (55.2)	8 (30.8)	45 (47.9)	47 (59.5)
Other^d^	9 (1.4)	0	2 (2.1)	2 (2.5)
Missing	2 (0.3)	0	0	0
Distance to screening center^e^				
≤Median	301 (47.7)	14 (53.8)	45 (47.9)	40 (50.6)
>Median	325 (51.5)	12 (46.2)	48 (51.1)	39 (49.4)
Missing	5 (0.8)	0	1 (1.1)	0
Median household income^a^^,^^f^				
≤Median	309 (49.0)	15 (57.7)	52 (55.3)	44 (55.7)
>Median	322 (51.0)	11 (42.3)	42 (44.7)	35 (44.3)
ADI state rank^g^				
≤Median	362 (57.4)	14 (53.8)	40 (42.6)	44 (55.7)
>Median	231 (36.6)	11 (42.3)	50 (53.2)	31 (39.2)
Missing	38 (6.0)	1 (3.8)	4 (4.3)	4 (5.1)
Type of referring physician^a^^,^^b^				
Pulmonology, thoracic oncology, radiology or surgery	102 (16.2)	4 (15.4)	20 (21.3)	24 (30.4)
Other^h^	529 (83.8)	22 (84.6)	74 (78.7)	55 (69.6)
Expected follow-up examination				
Before COVID-19	595 (94.3)	25 (96.2)	89 (94.7)	69 (87.3)
During COVID-19 pause	8 (1.3)	0	1 (1.1)	2 (2.5)
After COVID-19 pause	28 (4.4)	1 (3.8)	4 (4.3)	8 (10.1)

Abbreviations: ADI, Area Deprivation Index; CCI, Charlson Comorbidity Index; Lung-RADS, Lung CT Screening Reporting & Data System.

^a^ Variable was adjusted for in experiment 2 (ie, significant baseline factors from experiment 1).

^b^ *P* < .05 from the χ^2^ test.

^c^ Subcategories were American Indian or Alaska Native, Native Hawaiian or Pacific Islander, more than 1 race, or racial and ethnic group not otherwise stated.

^d^ Subcategories were Veterans Health Administration, self-pay, and other insurance not specified.

^e^ Median distance to screening center was 5.48 miles.

^f^ Median household income was $74 011.

^g^ Median ADI state rank was 3.

^h^ Subcategories were family medicine, general internal medicine, and obstetrics and gynecology.

For patients with a negative screening result at baseline, results from the GEE model suggested that the odds of being nonadherent to the Lung-RADS recommendations at the second screening increased in the group with unchanged negative screening results (AOR, 1.38; 95% CI, 1.12-1.69) but decreased in patients in the upgraded category (AOR, 0.29; 95% CI, 0.14-0.60) (Table 4). For those with a positive baseline screening result, the odds of being nonadherent at the following screening with a negative result increased (AOR, 5.08; 95% CI, 1.28-20.1). There was no significant change in adherence in the group with unchanged positive screening results at the second screening. In addition, no significant difference in adherence at the third screening was found across the 4 subgroups.

**Table 4.** Summary of Findings From Generalized Estimating Equations Analysis of Nonadherence to Lung-RADS Recommendations Measured Over Time Among 830 Patients in Experiment 2

Comparison of interest	Nonadherence to T1 Lung-RADS recommendations		Nonadherence to T2 Lung-RADS recommendations	
	AOR (95% CI)^a^	*P* value	AOR (95% CI)^a^	*P* value
Baseline Lung-RADS score of 1 or 2				
Unchanged subsequently vs T0	1.38 (1.12-1.69)	.002	1.17 (0.90-1.52)	.23
Upgraded subsequently vs T0	0.29 (0.14-0.60)	<.001	0.44 (0.19-1.01)	.054
Baseline Lung-RADS score of 3 or 4				
Unchanged subsequently vs T0	1.81 (0.62-5.22)	.28	1.34 (0.16-10.9)	.78
Downgraded subsequently vs T0	5.08 (1.28-20.1)	.02	6.99 (0.66-74.1)	.11

Abbreviations: AOR, adjusted odds ratio; Lung-RADS, Lung CT Screening Reporting & Data System; T0, first screening time point; T1, second screening time point; T2, third screening time point.

^a^ Adjusted baseline variables included baseline Lung-RADS score, family history of lung cancer, educational level, median household income, age-adjusted Charlson Comorbidity Index score, and type of referring physician.

## Discussion

As the volume of patients participating in clinical LCS practices increases, the challenge of addressing low adherence to Lung-RADS recommendations is magnified as observed among patients with negative screening results in this study. Identifying factors associated with nonadherence may help resource-constrained health systems to direct targeted outreach to patients who are at a high risk of nonadherence and thus likely to receive the greatest benefit from targeted interventions. Appointment reminders and/or LCS educational materials sent to patients by mail or via patient health portals in the electronic medical record as well as reinforcement of LCS-related benefits by the screening program are possible interventions to mitigate nonadherence.

Our findings that Lung-RADS scores and the type of referring physician were associated with patient nonadherence to baseline Lung-RADS recommendations aligned with previous studies.^15,16,17^ The baseline Lung-RADS score was the most important variable when estimating whether a patient would be adherent in returning for their initial follow-up screening examination. Patients with a negative baseline screening result were at high risk for nonadherence. A study by Wildstein et al^18^ found that a higher educational level (eg, at least a college degree) was associated with annual adherence to LCS, although the study was conducted prior to the release date of the Lung-RADS recommendations. Our study found that a family history of lung cancer, comorbidity (high vs low CCI score), and lower income were factors significantly associated with nonadherence at the first follow-up, a finding that, to our knowledge, has not been previously reported in LCS literature. These factors have been previously studied in colorectal and breast cancer screening^19,20,21^ but with sometimes conflicting results, as in the case of medical comorbidity.^19,20^ As such, further investigation on the clinical significance of these factors is necessary.

The major contribution of this study lies in the identification of changes in Lung-RADS scores as an important factor associated with nonadherence to LCS across multiple screening time points. Our analysis provides insights into which groups of patients may be more likely to be nonadherent in subsequent screening examinations. For example, if patients have had consecutive screenings with negative results, their adherence may diminish over time. In this study, individuals in this group tended to be younger at baseline and referred by physicians from nonpulmonary or nonthoracic departments. These observations may help inform which patients are at highest risk of nonadherence to annual screening, which can delay the diagnosis of lung cancer.^7,22^ Of note, cancers first observed at incidence screenings tend to be faster growing and more aggressive than those identified at prevalence screens,^7^ a finding indicating the importance of adherence to follow-up recommendations. In the present study, the GEE model results suggest that patients with a positive baseline screening result followed by a negative screening result may also need assistance in maintaining adherence at the first annual screening (ie, nonadherence increased over time). However, further investigation is needed given the wide 95% CI. Our findings regarding changes in adherence as patients underwent subsequent screenings underscore the need for screening programs to provide ongoing patient education and reminders, facilitate adherence by providing screening locations near the patient, and minimize patient inconvenience through timely scheduling and efficient patient throughput.

In the future, the findings of this study may be incorporated into a temporal model that helps evaluate adherence status at each screening time point, adding time-varying variables into the temporal model to achieve better performance by considering the changes in patients’ health status at each screening (eg, age-adjusted CCI score, smoking status, and insurance status). Finally, the use of specific types of outreach intervention (eg, reminders, consultations, and educational materials) to improve adherence will vary based on the underlying reason why an individual may be nonadherent. While reminders and educational outreach have been helpful in other screening contexts,^23,24^ a greater understanding of the psychological, cognitive, social, and health care practitioner factors associated with screening adherence may be essential to optimize outreach interventions. Further studies that explicitly examine these factors are needed.

### Limitations

This study has limitations. Several potential risk factors were not considered in our investigation due to a lack of data. Carter-Harris et al^25^ proposed additional important factors associated with patient behavior toward LCS, including patient psychological, cognitive, social, and environmental factors and health care practitioner recommendations. These variables were previously shown to be associated with patient behaviors toward screening for lung or other types of cancer.^26,27,28,29,30,31,32,33^ Unlike immutable factors, such as race and ethnicity, psychological and cognitive factors can change over time, providing opportunities for outreach interventions. Other factors associated with cancer screening rates are social determinants of health.^34,35^ Moreover, it was not possible to track patients who had permanently moved but continued LCS at outside institutions. The factors that we assessed were limited to data elements that were captured routinely in the medical records. Future work is needed to evaluate other life circumstances (eg, personal, such as health [eg, had other medical issues, so LCS was not a priority], family, and socioeconomic), professional activities (eg, workload), and social and environmental factors (eg, childcare and family responsibilities) that might affect adherence.

The lack of primary care physician involvement may be another major factor associated with patients’ adherence behaviors in LCS. Primary care physicians may be less familiar with LCS, its relative risks and benefits, and eligibility requirements for reimbursement compared with other cancer screening examinations. Although annual review of preventive health measures is inherent to primary care, LCS is nascent in practice, and there are myriad reasons why primary care referrals may be associated with less adherence. Compared with other preventive measures, LCS requires a greater time commitment for shared decision-making, smoking cessation counseling, and formal documentation. Our study only examined a high-level variable to distinguish primary care and subspecialty referrals, which cannot capture nuances of physician awareness or practice constraints.

## Conclusions

In this cohort study, we identified factors associated with patient nonadherence to Lung-RADS recommendations across 3 screening time points. We showed that the Lung-RADS score at baseline was the most important factor associated with nonadherence at the initial follow-up screening. Patients with consecutive negative screening results were at the greatest risk of being nonadherent to a subsequent screening. Our study provides evidence that may be used as the basis of a decision-support tool to estimate nonadherence across multiple time points and inform future outreach interventions designed to improve patient adherence to LCS.

## Notes

Supplement 1. **eAppendix 1.** Supplemental Methods and Results **eAppendix 2.** Performance of Machine Learning Classifiers Trained Using Identified Predictors **eFigure 1.** Examples of Determining Patient Adherence Statuses to Lung-RADS Recommendations **eFigure 2.** Overall Pipeline of the Experiment Described in eAppendix 1 **eTable 1.** Comparison of Observed Baseline Characteristics Between Included Patients and Excluded Patients for Experiment 1 **eTable 2.** Possible Scenarios of Longitudinal Patterns in Lung-RADS Scores **eTable 3.** Validation Performance of Machine Learning Models Using Repeated (n = 5) 10-fold Cross-Validation **eTable 4.** Summary of the Number of Patients Enrolled Each Year and Their Mean Follow-Up Time

Supplement 2.
Data Sharing Statement
