# Supplementary material for: Factors Associated With Nonadherence to Lung Cancer Screening Across Multiple Screening Time Points
Source: JAMA Netw Open. 2023 May 25;6(5):e2315250. doi: 10.1001/jamanetworkopen.2023.15250 (PMC10214032; doi:10.1001/jamanetworkopen.2023.15250)
Supplement: Supplement 1. — eAppendix 1. Supplemental Methods and Results eAppendix 2. Performance of Machine Learning Classifiers Trained Using Identified Predictors eFigure 1. Examples of Determining Patient Adherence Statuses to Lung-RADS Recommendations eFigure 2. Overall Pipeline of the Experiment Described in eAppendix 1 eTable 1. Comparison of Observed Baseline Characteristics Between Included Patients and Excluded Patients for Experiment 1 eTable 2. Possible Scenarios of Longitudinal Patterns in Lung-RADS Scores eTable 3. Validation Performance of Machine Learning Models Using Repeated (n = 5) 10-fold Cross-Validation eTable 4. Summary of the Number of Patients Enrolled Each Year and Their Mean Follow-Up Time [file jamanetwopen-e2315250-s001.pdf]

Supplementary Online Content

Lin Y, Liang LJ, Ding R, Prosper AE, Aberle DR, Hsu W. Factors associated with nonadherence to lung cancer screening across multiple screening time points. *JAMA Netw Open*. 2023;6(5):e2315250. doi:10.1001/jamanetworkopen.2023.15250

eAppendix 1. Supplemental Methods and Results

eAppendix 2. Performance of Machine Learning Classifiers Trained Using Identified Predictors

eFigure 1. Examples of Determining Patient Adherence Statuses to Lung-RADS Recommendations

eFigure 2. Overall Pipeline of the Experiment Described in eAppendix 1

eTable 1. Comparison of Observed Baseline Characteristics Between Included Patients and Excluded Patients for Experiment 1

eTable 2. Possible Scenarios of Longitudinal Patterns in Lung-RADS Scores

eTable 3. Validation Performance of Machine Learning Models Using Repeated (n = 5) 10-fold Cross-Validation

eTable 4. Summary of the Number of Patients Enrolled Each Year and Their Mean Follow-Up Time

This supplementary material has been provided by the authors to give readers additional information about their work.

© 2023 Lin Y et al. *JAMA Network Open*.

eAppendix 1. Supplemental Methods and Results

Supplemental Methods

Identification of screen-eligible patients: The primary care providers or pulmonologists determined if a patient was eligible to participate in lung cancer screening (LCS), then referred them to the LCS program. Health professionals in the Screening Program provided shared decision making (including smoking cessation counseling) if this had not been documented in the electronic medical record.

Intervention for adherence: After each screen, the patients would receive reminders for the follow-up examination from two sources, 1) their primary care providers or pulmonologists and 2) staff of our LCS program. However, patients who decided not to come back for screening were not contacted to record the reasons for non-adherence.

Documentation of time data: The completion date of each LDCT was retrieved from the Integrated Diagnostic Lung database. If a follow-up screen was completed, then the difference between the two LDCT dates was used to determine adherence; if a follow-up screen was not completed, then we would wait 15 (Lung-RADS 1 and 2), 9 (Lung-RADS 3), 5 (Lung-RADS 4A), and 3 (Lung-RADS 4B/X) months before categorizing the patient as non-adherent.

Determination of screening time points: The three screening time points were the actual dates of the patient’s first three low-dose CT screening examinations. T0: baseline/first screen; T1: second screen; T2: third screen.

Supplemental Results

Rate of non-adherence among patients with lung cancer: Among patients diagnosed with lung cancer, 11% (9/81) had not adhered to screening recommendation.

© 2023 Lin Y et al. *JAMA Network Open*.

eAppendix 2: Performance of Machine Learning Classifiers Trained Using Identified Predictors

Methods

We trained and evaluated five machine learning models to predict patient non-adherence to baseline Lung-RADS recommendations (see **eFigure 2** in the Supplement). The inputs into the models were significant (i.e., z test, two-sided p-value<0.05) baseline predictors from Experiment 1. This experiment aimed to validate whether these predictors could correctly classify patients who were non-adherent to baseline Lung-RADS recommendations. Logistic regression, random forest, support vector machine, naïve Bayes, and XGBoost were trained using data without missing values in all input variables. Naïve Bayes was also trained using data with missing values left as-is. We used nested 10-fold cross-validation, repeated five times to select the best models based on the primary evaluation metric, recall/sensitivity, in the validation sets, which reflected the model's ability to identify truly non-adherent patients correctly. The best-performing model was then retrained and tested on the hold-out test cases (n=278 from data with no missing values in significant predictors from Experiment 1). Secondary evaluation metrics included precision/positive predictive value (PPV), accuracy, and area under the receiver operating characteristic curve (ROC-AUC).

Results

Among 2496 eligible patients, 278 with no missing values in significant predictors from Experiment 1 were used as the hold-out test set. Of the remaining 2218 patients, 300 patients had missing values in some significant predictors from Experiment 1, leaving 1918 patients with no missing values in the significant predictors from Experiment 1. 2218 (with missing values) and 1918 (without missing values) patients were used for cross-validation (see **eFigure 2** in the Supplement). The inputs into the machine learning models were the six significant baseline predictors from Experiment 1. Model performance on the validation sets of the five machine learning models is shown in **eTable 3**. Most models achieved greater than 90% recall/sensitivity and similar performance in other evaluation metrics. The final retrained logistic regression model achieved recall/sensitivity: 0.939, precision/PPV: 0.712, accuracy: 0.716, and ROC-AUC: 0.667 on the hold-out test cases.

Discussion

We show that machine learning models trained on significant predictors identified in Experiment 1 can capture most non-adherent patients (i.e., high recall/sensitivity), only missing 6% of non-adherent patients. Given that some predictors may not be routinely collected in medical records (e.g., income), the model that handles missing values (i.e., naïve Bayes) is useful for making classification when values of certain variables are missing. We should note that the analysis was influenced by the screening population that is seen at our institution; other institutions may identify specific predictors that affect adherence in their lung cancer screening population.

Limitation

Despite a high recall/sensitivity, the accuracy of the prediction models was around 70%, resulting in some patients who are likely to be adherent in practice being misidentified as having a high risk of non-adherence. In a targeted approach to adherence interventions, these individuals may receive unnecessary outreach; in this scenario, the negative impact is minimal to the patient but may divert critical resources away from other essential services.

© 2023 Lin Y et al. *JAMA Network Open*.

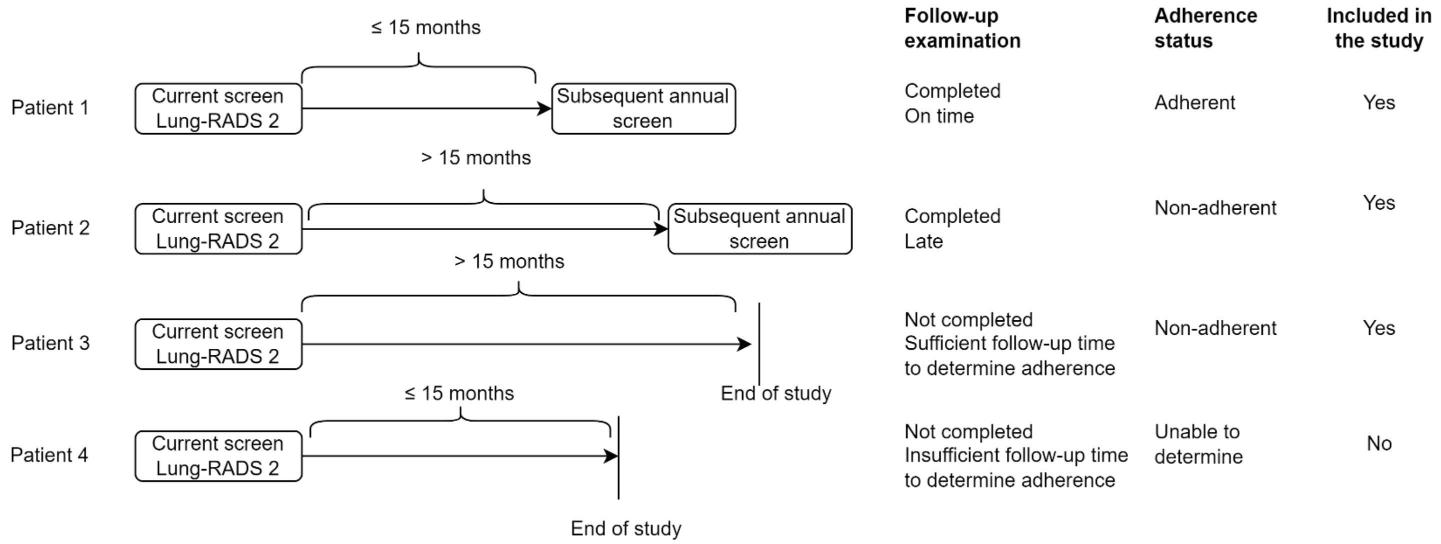

eFigure 1. Examples Of Determining Patient Adherence Statuses to Lung-RADS Recommendations. All patients were assumed to have had a current Lung-RADS 2 screen. Adherence was defined as completion of the subsequent annual screen within 15 months from the current screen. Lung-RADS: Lung CT Screening Reporting & Data System.

© 2023 Lin Y et al. *JAMA Network Open*.

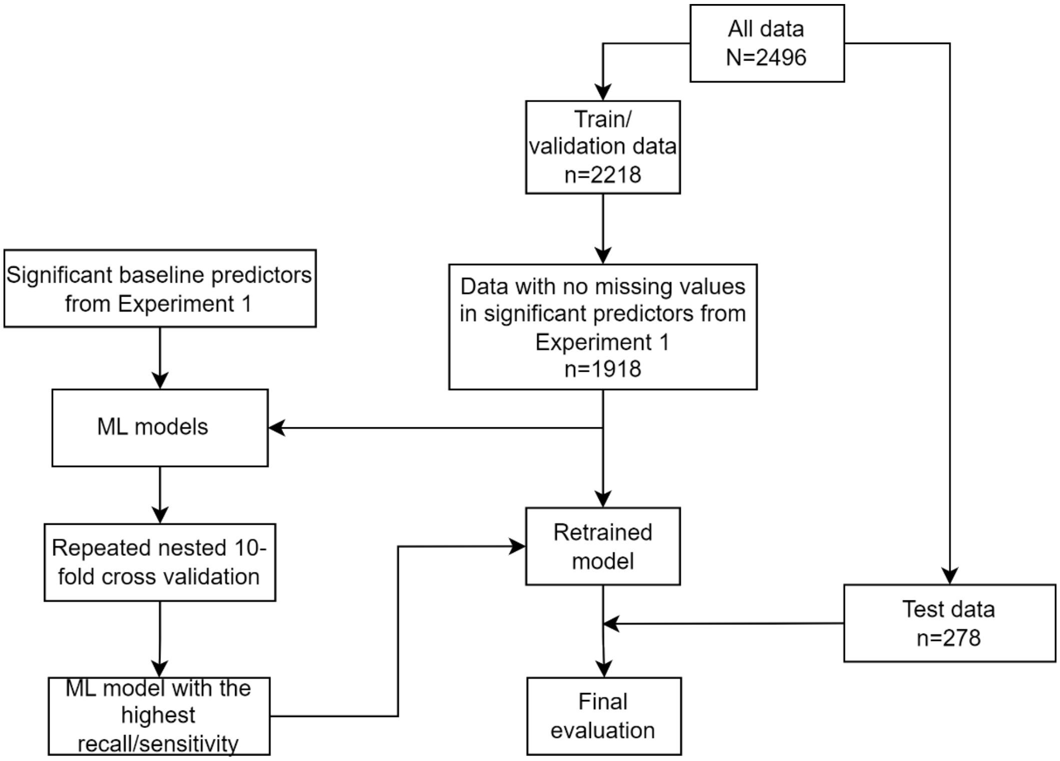

eFigure 2. Overall Pipeline of the Experiment Described in eAppendix 1. Using ML models to predict patient adherence to baseline Lung-RADS recommendations. Lung-RADS: Lung CT Screening Reporting & Data System. ML: Machine learning; Note: Test data only included patients with no missing significant predictors from Experiment 1.

© 2023 Lin Y et al. *JAMA Network Open*.

eTable 1. Comparison of Observed Baseline Characteristics Between Included Patients and Excluded Patients for Experiment 1.

	Included	Excluded	p
**n (%)**	**1979**	**517**	
Lung-RADS			
1-2	1660 (83.9)	433 (83.8)	0.997
3-4	319 (16.1)	84 (16.2)	
Age in years			
<65	868 (43.9)	207 (40.0)	0.130
>=65	1111 (56.1)	310 (60.0)	
Sex			
Female	803 (40.6)	207 (40.0)	0.864
Male	1176 (59.4)	310 (60.0)	
Family history of lung cancer			
Yes	466 (23.5)	92 (17.8)	0.006
No	1513 (76.5)	425 (82.2)	
Age adjusted CCI			
Low (0-1)	287 (14.5)	68 (13.2)	0.477
Intermediate or high (>1)	1692 (85.5)	449 (86.8)	
Expected follow-up exam			
Pre-COVID	1468 (74.2)	381 (73.7)	0.555
During COVID pause	53 (2.7)	10 (1.9)	
Post-COVID pause	458 (23.1)	126 (24.4)	

[a] p: two-sided p values of Chi-square tests.

Lung-RADS: Lung CT Screening Reporting & Data System; CCI: Charlson Comorbidity Index.

© 2023 Lin Y et al. *JAMA Network Open*.

eTable 2. Possible Scenarios of Longitudinal Patterns in Lung-RADS Scores.

Category	Lung-RADS score		
	Time point 1	Time point 2	Time point 3
Unchanged	1 or 2	1 or 2	NA
	1 or 2	1 or 2	1 or 2
	3 or 4	3 or 4	NA
	3 or 4	3 or 4	3 or 4
Downgraded	3 or 4	1 or 2	NA
	3 or 4	1 or 2	1 or 2
	3 or 4	3 or 4	1 or 2
Upgraded	1 or 2	3 or 4	NA
	1 or 2	3 or 4	3 or 4
	1 or 2	1 or 2	3 or 4

[a] When a Lung-RADS score is NA, it can either be that the recommended date of the patient's third screen was scheduled after the last follow-up date of this study or the patient had a third screen but with insufficient follow-up time to determine adherence status to the third Lung-RADS recommendation.

Lung-RADS: Lung CT Screening Reporting & Data System, NA: not available.

© 2023 Lin Y et al. *JAMA Network Open*.

eTable 3. Validation Performance of Machine Learning Models Using Repeated (n = 5) 10-fold Cross-Validation.

Model/Metric (SD)	Recall/sensitivity	Precision/PPV	Accuracy	AUC
**Complete case training/validation data n=1918**				
Logistic regression	0.939 (0.027)	0.682 (0.032)	0.679 (0.030)	0.662 (0.039)
Naïve Bayes	0.916 (0.029)	0.691 (0.034)	0.682 (0.033)	0.662 (0.039)
XGBoost	0.912 (0.028)	0.692 (0.030)	0.682 (0.027)	0.656 (0.038)
SVM	0.909 (0.026)	0.694 (0.032)	0.684 (0.030)	0.622 (0.047)
Random forest	0.896 (0.031)	0.688 (0.033)	0.670 (0.031)	0.626 (0.039)
**All training/validation data with missing values n=2218**				
Naïve Bayes	0.919 (0.019)	0.694 (0.027)	0.686 (0.023)	0.590 (0.020)

Input variables: significant baseline predictors from Experiment 1 including Lung-RADS, family history of lung cancer, education level, median household income, age-adjusted Charlson Comorbidity Index, and type of referring physician.

SD: standard deviation; PPV: positive predictive value; AUC: area under the receiver operating characteristics curve; SVM: support vector machine.

© 2023 Lin Y et al. *JAMA Network Open*.

eTable 4. Summary of the Number of Patients Enrolled Each Year and Their Mean Follow-Up Time (N=2496, patients with up to three adherence status assessments).

Year	2013	2014	2015	2016	2017	2018	2019	2020	2021
No. patients enrolled	3	106	329	381	410	481	420	311	55
Mean follow-up time in years	3.75	2.99	2.16	2.08	1.99	1.65	1.4	1.16	0.71

© 2023 Lin Y et al. *JAMA Network Open*.
