# Supplementary material for: Factors Associated With Nonadherence to Lung Cancer Screening Across Multiple Screening Time Points
Source: JAMA Netw Open. 2023 May 25;6(5):e2315250. doi: 10.1001/jamanetworkopen.2023.15250 (PMC10214032; doi:10.1001/jamanetworkopen.2023.15250)
Supplement: Supplement 2. — Data Sharing Statement [file jamanetwopen-e2315250-s002.pdf]

Data Sharing Statement

Lin. Factors Associated With Nonadherence to Lung Cancer Screening Across Multiple Screening Time Points. *JAMA Netw Open*. Published May 25, 2023. doi:10.1001/jamanetworkopen.2023.15250

Data
Data available: No
